# From Cytoskeletal Remodeling to Oocyte Quality: The Emerging Role of Mechanics

**DOI:** 10.1002/advs.76323

**Published:** 2026-06-26

**Authors:** Anastasia Shihabi, Rose Bulteau, Lucie Barbier, Marie‐Emilie Terret

**Affiliations:** ^1^ Center For Interdisciplinary Research in Biology (CIRB) College De France CNRS INSERM Université PSL Paris France

**Keywords:** actomyosin networks, assisted reproductive technologies (ART), female fertility, meiotic maturation, oocyte mechanical properties, oocyte quality

## Abstract

Oocyte quality is essential for successful fertilization and embryonic development. At the end of mammalian oogenesis, oocytes undergo two highly asymmetric meiotic divisions that preserve maternal reserves while generating a haploid gamete. These divisions rely on dynamic cytoplasmic and cortical actomyosin networks. The mechanical properties of the oocyte, shaped by the remodeling of these networks, are critical regulators of oocyte morphogenesis, controlling cytoplasmic organization, spindle positioning, chromosome segregation, and cytokinesis. Meiotic errors, which increase with maternal age, compromise fertility and embryonic development. In parallel, the growing use of assisted reproductive technologies, including oocyte freezing for fertility preservation, has intensified the need for reliable markers of oocyte quality. Beyond conventional morphological assessment, oocyte mechanical properties have recently emerged as promising indicators of developmental competence, as naturally occurring mechanical defects can impair oocyte quality. This review discusses the molecular and cellular pathways regulating oocyte mechanics in relation to actomyosin reorganization, their roles during oocyte divisions, their alteration in pathological contexts, and their potential clinical applications as markers in reproductive medicine.

## Graphical abstract

Mechanical properties of the oocyte are increasingly recognized as critical determinants of meiotic success and developmental potential. How actomyosin remodeling controls oocyte mechanics, how these properties are altered in pathological conditions, and how they could serve as markers in reproductive medicine are examined.

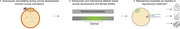

## 1 Introduction

Oocytes, the female gametes, are essential for sexual reproduction, allowing the formation of a new individual through fertilization by the sperm, the male gamete. In mammals, oocytes are produced through a long process known as oogenesis, which begins during embryonic development and proceeds cyclically from puberty to menopause through the key steps of oocyte growth and meiotic maturation. During growth, the oocyte arrested in prophase I accumulates maternal reserves (mRNAs, proteins, organelles) essential for supporting both meiotic maturation and embryonic development [1, 2, 3]. During maturation, the oocyte completes the first meiotic division (meiosis I) and becomes arrested at metaphase of the second meiotic division (meiosis II). Meiosis completion then occurs if fertilization takes place, triggering anaphase and exit from the second meiotic division. The two meiotic divisions are highly asymmetric in size, following each other without an intermediate DNA replication step, in order to obtain a haploid cell while minimizing the loss of maternal reserves accumulated in the oocyte during its growth [2, 3, 4]. The oocyte achieves these asymmetric divisions thanks to the off‐center positioning of the spindle in meiosis I and II. In meiosis I, the spindle migrates from the oocyte center where the nucleus was located to the cortex [5, 6], and reassembles directly under the cortex in meiosis II.

Meiotic divisions are prone to errors, resulting in a basal rate of poor‐quality oocytes that increases with maternal age and has deleterious consequences for fertility and embryonic development [7, 8, 9, 10, 11, 12, 13, 14]. Errors in meiotic division stem, among other things, from several intrinsic characteristics of oocytes. Notably, oocytes are long‐lived, large cells that lack canonical centrosomes [15, 16], thus relying on alternative microtubule‐independent strategies to position their spindle through actomyosin networks. These networks include cytoplasmic and cortical actomyosin structures. Both are highly dynamic during meiotic maturation and undergo extensive remodeling. Their remodeling leads to major changes in the mechanical properties of the oocyte, which play a crucial role in regulating cytoplasmic organization, spindle migration, chromosome segregation, and cytokinesis, all key determinants of oocyte quality. Mechanical properties could therefore be considered as a direct readout of oocyte quality, reflecting the actomyosin architecture, which is tightly linked to key processes ensuring correct oocyte morphogenesis.

Indicators of oocyte quality are highly relevant for oocyte selection in assisted reproductive technologies (ART), including oocyte freezing for fertility preservation, where the assessment of oocyte quality remains challenging and limits the success rates of these procedures. Over the past decade, an increasing number of women and couples have postponed childbearing [17], leading to increased use of ART to conceive. In a clinical context, oocyte quality is primarily assessed based on morphological criteria. While morphological assessment allows the exclusion of low‐quality oocytes, it does not necessarily identify those with the highest developmental potential [18]. Thus, in recent years, mechanical properties have emerged as potential markers for assessing oocyte quality and improving ART outcomes [19, 20].

This review aims to examine the mechanical properties of mammalian oocytes as biophysical determinants of female fertility. It first presents the reorganization of actomyosin networks in relation to oocyte mechanical properties, and focuses on the key steps of meiotic maturation regulated by the interplay between actomyosin networks and mechanical properties. It then discusses mechanical defects in pathological contexts and their implications for female fertility. Finally, it explores the potential of mechanical properties as markers of oocyte quality in a clinical context, the different techniques used to assess these properties and their applicability. This review is not intended to be exhaustive but rather to highlight key studies that have advanced our understanding of these processes.

## 2 Actomyosin Organization and Mechanical Landscape of the Oocyte

**FIGURE 1:**
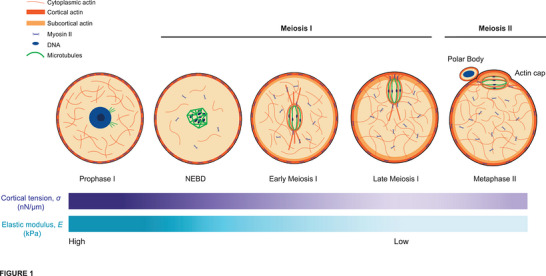
Mechanical landscape and actomyosin organization in mouse oocytes during meiotic maturation. Oocytes in prophase I have a dense cytoplasmic actin meshwork and a thin actin cortex enriched in active Myosin II, resulting in a high cortical tension and a high elasticity. After meiosis I resumption, marked by nuclear envelope breakdown (NEBD), the actin meshwork dismantles and reforms from mid‐meiosis I, including an F‐actin cage surrounding the bipolar spindle. In parallel, a subcortical actin network is progressively nucleated, chasing Myosin II from the cortex, resulting in a decrease in cortical tension and elasticity. Myosin II relocates in the cytoplasm and on the spindle, particularly at the spindle poles, allowing spindle migration and a first asymmetric division in size. The oocyte enters meiosis II and arrests in metaphase II, characterized by an actin cap above the spindle with higher stiffness compared to the rest of the oocyte.

Actomyosin networks organize cell shape and contribute to cell mechanical properties [21]. Strikingly, oocytes display highly dynamic spatiotemporal reorganization of actomyosin during their maturation, which in turn modulates their mechanical properties (Figure 1).

### 2.1 Actomyosin Networks in Oocytes During Meiotic Maturation

At the end of their growth in prophase I, mouse oocytes contain a dense cytoplasmic F‐actin network (Figure 1). This cytoplasmic F‐actin network is nucleated by Formin 2 [22, 23, 24, 25], which cooperates with Spire 1 and Spire 2 [26]. Spire proteins are localized on Rab‐11a‐positive vesicles transported by myosin Vb along actin filaments, and in turn recruit Formin 2 to promote actin filament assembly [26, 27]. After meiosis I resumption and nuclear envelope breakdown (NEBD), this network is dismantled [28] (Figure 1). Progressively, a new cytoplasmic F‐actin network, comparable to that observed in prophase I, reassembles, including an F‐actin cage surrounding the spindle (Figure 1). A similar cytoplasmic F‐actin meshwork has been described in human oocytes [29, 30], although the underlying molecular mechanisms remain less characterized.

In parallel with the remodeling of cytoplasmic actin, the actomyosin cortex also undergoes significant changes during meiosis I. In prophase I, the oocyte cortex is thin (less than 1 µm). After NEBD, activation of the Mos‐MAPK (Mitogen‐Activated Protein Kinase) pathway leads to activation of the Arp2/3 complex, progressively nucleating a subcortical F‐actin network (Figure 1) that thickens up to 4 µm prior to polar body extrusion (PBE) [31, 32]. Concomitantly, Myosin II, initially located at the cortex in prophase I, is excluded from the cortex after NEBD and relocates to the cytoplasm and spindle, particularly at spindle poles [25, 31, 33] (Figure 1). Myosin II is displaced from the cortex by subcortical actin nucleation [31, 33], which may create steric hindrance, pushing Myosin II away from the cortex, a hypothesis supported by findings in somatic cells [34]. After meiosis I, the oocyte arrests in metaphase of meiosis II. At this stage, oocytes are polarized, with a region above the second meiotic spindle enriched in actin and active Myosin II [5, 35, 36, 37, 38].

### 2.2 Cellular Mechanical Properties

At the subcellular level, actomyosin networks can generate forces that drive cytoplasmic stirring and intracellular transport [39, 40, 41, 42, 43, 44, 45]. At the cellular level, the architecture of these networks contributes to the overall mechanical properties that characterize the ability of the cell to deform or resist deformation when subjected to external forces. Three main parameters are commonly used to describe these mechanical properties: elasticity, viscosity and cortical tension. These descriptors rely on simple and ideal cases that treat the cell as a continuous material.

As a first approximation, a cell can be considered a pure elastic solid. Elasticity refers to the ability of a cell to deform in response to an applied force and to return to its original state once the force is removed [46]. It is quantified by the Young's modulus (*E*), an intrinsic property that is independent of the material's size, shape, mass and boundary conditions [47]. However, living cells also dissipate energy and therefore cannot be described solely as pure elastic solids. They can exhibit fluid‐like behavior and are often approximated as Newtonian fluids. In this framework, resistance to flow is characterized by viscosity, and an effective cellular viscosity (*η*) is commonly defined [46]. The Young's modulus of somatic cells typically ranges from approximately 100 Pa to 10 kPa, while their viscosity is generally on the order of 100 to 1000 Pa.s [48]. For oocytes, the Young's modulus and the viscosity are generally within the same range as that of somatic cells [20, 49, 50, 51, 52, 53]. However, the measured values strongly depend on the experimental technique used, as different methods probe either the global mechanical properties of the cell or more localized mechanical responses (see Section 2.3). In many experimental situations, cells exhibit mechanical behaviors that lie between those of an elastic solid and a purely viscous fluid. Cells are therefore more accurately described as viscoelastic materials exhibiting time‐dependent mechanical responses.

While elasticity and viscosity are bulk properties characterizing internal cell deformation, cortical tension is a surface property. It is commonly modeled by analogy with a liquid droplet exhibiting surface tension. In this context, surface tension is defined as the energy required to increase the surface area by one unit area. In cells, cortical tension (*σ*) is set by the spatiotemporal organization and contractility of the cell cortex [54]. Typical values of cortical tension for somatic cells range from approximately 10^−3^ to 10^−1^ nN µm^−1^ [55, 56, 57]. For oocytes, cortical tension is generally within the same range, although higher values have been reported during early stages of meiotic maturation, reaching about 1 nN µm^−1^ [31, 52, 58].

### 2.3 Dynamic Regulation of Oocyte Mechanical Properties During Meiotic Maturation

The displacement of Myosin II from the cortex after NEBD, due to cortical actin nucleation and subsequent cortical actin thickening, results in a decrease in cortical tension in meiosis I [31, 58], as well as a decrease in elasticity [52] (Figure 1). As a result, mouse oocytes with a thick actin cortex are softer, whereas those with a thin cortex are stiffer [52]. Interestingly, this decrease in cortical tension and elasticity does not occur in meiosis I in human oocytes, but instead in meiosis II. Accordingly, the cortex of human oocytes does not thicken during meiosis I [52], suggesting species‐specific regulation of cortical actin. Finally, metaphase II‐arrested oocytes are polarized, with a stiffer domain at the cortex directly above the spindle [58, 59, 60] (Figure 1).

Oocyte viscosity during meiotic maturation is more difficult to interpret, as it strongly depends on the measurement method and thus on the spatial scale probed. It can be assessed from the micrometer scale, using optical tweezers [41, 61], mean squared displacement measurement of fluorescent beads [42] and atomic force microscopy (AFM) [62], to the whole‐cell scale using imaging‐based methods [63] and significant cell deformation approaches such as micropipette aspiration and microfluidics [49, 62]. In mouse oocytes, at the micrometer scale in the cytoplasm, the viscous modulus decreases between prophase I and metaphase I [42]. At these stages, active diffusion of Rab‐11a‐positive actin‐coated vesicles, driven by myosin Vb, promotes fluidization of the cytoplasm, facilitating intracellular transport of organelles [41, 42]. At the micrometer scale in the cortex, cortical viscosity remains relatively constant during meiosis I (Figure 1), whereas viscosity decreases globally at the oocyte scale [49]. In contrast, in human oocytes, cortical viscosity increases from prophase I to meiosis II [52], and global viscosity at the oocyte scale also increases from prophase I to meiosis II, as measured using the persistence of the injection funnel after intracytoplasmic sperm injection (ICSI), an ART technique, as a proxy for effective viscosity [64].

Thus, oocyte mechanical properties are tightly regulated during meiotic maturation in response to dynamic spatiotemporal changes in cytoskeleton organization. These changes arise from molecular‐scale regulatory processes that drive cytoskeletal remodeling and cause cell‐scale mechanical modifications during maturation.

## 3 Key Processes Mechanically Regulated by Actomyosin Networks in Oocytes

### 3.1 Spindle Off‐Center Positioning in Meiosis I

In prophase I, mouse oocytes contain a dynamic cytoplasmic F‐actin network (Figure 1) that creates a pressure gradient and fluidifies the cytoplasm, thereby centering the nucleus [41]. At this stage, the oocyte shows no sign of polarity [65, 66]. After meiosis I resumption, the spindle forms in the central region of the oocyte, where the nucleus was previously located, transitioning from a ball to an elliptical shape in the absence of mechanical constraints thanks to the dismantlement of the cytoplasmic actin meshwork [6, 28] (Figure 1). Mid‐meiosis I, when the spindle is bipolar, a dynamic cytoplasmic F‐actin network, including an actin cage that surrounds the microtubule spindle, is progressively nucleated, together with a cortical actin thickening (Figure 1). Myosin II is displaced from the cortex and relocates to the cytoplasm and the spindle, where it can generate pulling forces that off‐center the spindle along its long axis toward the nearest cortical region [6, 25, 31, 33, 67]. This mechanism was recently confirmed by a study using isolated meiotic spindles [68]. The direction of migration is biased by the initial slight off‐centering of the spindle when it forms, due to the actin meshwork dismantlement and the consequent absence of an active centering mechanism [28], as well as by its pushing by local Formin 2‐dependent actin nucleation against organelles, resulting in one pole being closer to the cortex (the leading pole) [6, 69, 70, 71]. This generates a small imbalance in pulling forces generated by Myosin II, which are stronger at the pole closest to the cortex, thereby biasing spindle migration toward that cortical region. Following subcortical actin nucleation, the overlap between the cytoplasmic F‐actin meshwork and the subcortical network increases, likely providing a better substrate for Myosin II and thereby accelerating spindle migration [49]. This off‐center spindle positioning in meiosis I, required for asymmetric division, does not control the timing of anaphase. Indeed, disruption of F‐actin during meiosis I inhibits spindle migration, but anaphase occurs on time [6, 22, 23].

### 3.2 Meiosis I Cytokinesis and First Polar Body Extrusion

Toward the end of meiosis I, as the spindle approaches the cortex, an actomyosin cap forms above it [5, 67], marking the position of the future polar body extrusion [72]. Its formation depends on the close proximity of the chromosomes to the cortex [67, 73] and on the Arp2/3 complex [74]. A Ran‐GTP gradient centered around the chromosomes [75], and polo‐like kinase 1 (Plk1) located at the spindle poles during spindle migration [76], are essential for inducing actin cap formation through the recruitment of activators of the Arp2/3 complex, such as Rac, Cdc42 and N‐Wasp [66, 76, 77, 78]. RhoA and ROCK accumulate at the cortex closest to the spindle in mouse and porcine oocytes [79, 80] and are required for the recruitment of active Myosin II to the cortex in bovine [81] and mouse oocytes [82]. The actomyosin cap plays a dual role in first polar body extrusion. First, it generates actin flows that are converted into cytoplasmic flows [40], propelling the spindle toward the oocyte membrane and promoting cortical protrusion as anaphase progresses [83]. Second, it is essential for cytokinesis [6, 22, 75], as it enables formation of the cytokinesis furrow [23, 72, 82, 84], ensuring cytokinesis after anaphase I.

### 3.3 Meiosis II Arrest

**FIGURE 2:**
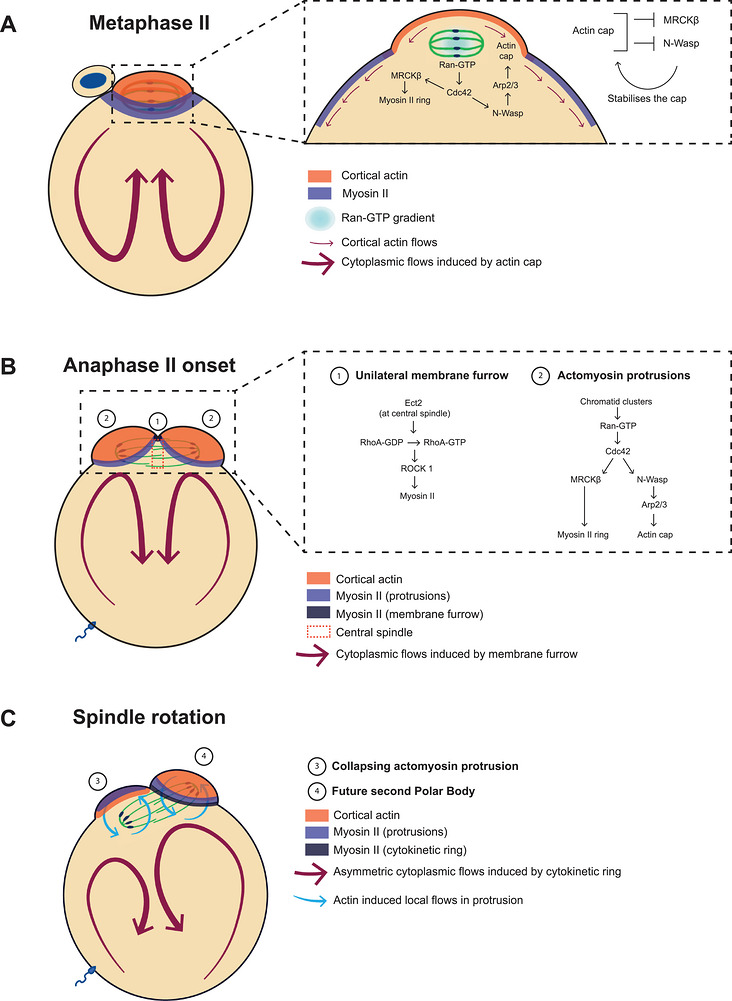
Actomyosin organization in metaphase II‐arrested mouse oocytes and reorganization triggered by fertilization. (A) In metaphase II‐arrested oocytes, the spindle is located under the cortex and triggers the formation of an actomyosin cap. This actomyosin cap is essential for spindle anchoring. The enlarged inset illustrates the molecular pathways responsible for actomyosin polarization and stabilization, inducing cytoplasmic flows in the oocyte. Inhibition of MRCKβ and N‐Wasp by the actin cap contribute to its stabilization. (B) Upon anaphase II onset, triggered by fertilization, a unilateral membrane furrow forms above the central spindle (1) and as chromosomes segregate, two novel actomyosin caps (2) form above each chromosome cluster. Myosin II is recruited to both structures, through different molecular pathways illustrated in the enlarged inset. The unilateral furrow induces reversed cytoplasmic flows in the oocyte. (C) After chromosome segregation, as the spindle rotates due to asymmetric cytoplasmic flows created by the ingression furrow, local actin flows emerge under the two novel actomyosin caps and maintain the chromosome clusters at the cortex. Nonetheless, DNA‐cortex distance increases for the internalized cluster during spindle rotation, which results in the collapsing of the actomyosin cap (3), while the remaining cap leads to cytokinesis and second polar body extrusion (4).

After the first polar body extrusion, the oocyte enters into meiosis II, reforms a spindle directly beneath the cortex, and arrests in metaphase II until fertilization. The spindle lies parallel to the cortex and remains anchored to it, promoting the formation of an actin cap, delineated by a ring of activated Myosin II [5, 35, 36, 37, 38, 73] (Figure 2A). This cortical polarity generates a stiffer domain directly above the spindle [58]. This actomyosin cap depends on the Ran‐GTP gradient centered on the chromosomes [73], as in meiosis I. In metaphase II, this gradient leads to the activation of Cdc42 above the spindle [77], enabling recruitment of the Arp2/3 complex through N‐Wasp [39] (Figure 2A). In addition, Cdc42 activation provides a spatial cue for recruitment of Myotonic dystrophy kinase‐related Cdc42‐binding kinase isoform beta (MRCKβ) at the cortex, leading to phosphorylation of myosin regulatory light chain (MRLC) and establishment of an active Myosin II ring [45] (Figure 2A). Myosin II contractility induces outward bulging of the membrane above the spindle in metaphase II, giving the cap its characteristic shape and stability [45, 82]. The cap also exerts a negative feedback on N‐Wasp and MRCKβ, contributing to its stabilization [45, 76] (Figure 2A).

Spindle anchoring depends on this actomyosin cap [35, 37, 39]. In the vicinity of the chromosomes, Rac is activated [66] and acts upstream of the Arp2/3 complex [85, 86], regulating spindle anchoring by promoting branched actin nucleation at the closest cortex. This actin nucleation maintains spindle positioning at the cortex through two mechanisms. First, it generates actin flows that induce cytoplasmic streaming, producing a net pressure on the spindle that pushes it toward the cortex (Figure 2A). Second, these cytoplasmic flows counteract the opposing forces caused by Myosin II‐driven contractions at the cap, which could push the spindle away from the cortex before anaphase [39]. Actin polarization is also observed in metaphase II‐arrested human oocytes [87], although further work is required to determine whether and how this polarization contributes to human oocyte development.

### 3.4 Anaphase II and Spindle Rotation

Fertilization triggers anaphase II and meiosis II exit. However, as the spindle is oriented parallel to the cortex in mouse oocytes, it must rotate during anaphase to enable extrusion of the second polar body. After anaphase II onset, the spindle midzone interacts with the cortex [43], inducing a RhoA‐dependent unilateral membrane furrow [44, 88] that overlays the anaphase spindle [38] (Figure 2B). This furrow is marked by localized activation of Ect2, enabling recruitment of Myosin II [88] through the central spindle/RhoA pathway [44] (Figure 2B). As chromosomes segregate, two Ran‐GTP gradients emerge from the separating chromatid clusters [44], recruiting Cdc42 above each cluster [44, 45, 77], resulting in two actomyosin caps overlying the segregated chromatids [43, 45] (Figure 2B). During anaphase II, the ingression of the unilateral membrane furrow enriched in Myosin II generates symmetrical reversed cytoplasmic flows [43, 44] (Figure 2B). To prevent spindle drifting from the cortex due to these reversed flows, the newly formed actomyosin caps stabilize the chromosomes through actin‐driven local flows at both caps [44] (Figure 2C). The furrow also defines a pivot point, which in combination with the actin‐driven local flows at both caps results in asymmetric reversed cytoplasmic flows [43, 44], thereby enabling spindle rotation (Figure 2C). In later stages of spindle rotation, the cortical protrusion above the most internalized chromatid cluster collapses, due to increased distance between the chromatids and the cortex [44] (Figure 2C).

## 4 Mechanical Defects and Implication for Female Fertility

Mechanical defects have been shown to exist naturally in mammalian oocytes [89] reflecting the inherent variability in oocyte mechanical properties [52] However, the origin of these mechanical defects remains unknown. As discussed above, the mechanical properties of the oocyte and the molecular pathways underlying them are associated with the actomyosin cytoskeleton and play a key role in oocyte meiotic maturation. Consequently, defects in actomyosin organization and mechanical properties can impair oocyte development and ultimately compromise female fertility.

### 4.1 Mechanotransduction, From Actomyosin Forces to Chromosomes

In prophase I, cytoplasmic forces are generated by the actin meshwork. Rab‐11a‐positive actin‐coated vesicles exert a pressure gradient that is higher at the cortex than at the center, due to a slight bias in vesicle speed and directionality toward the cortex. This pressure gradient propels the nucleus toward the oocyte center [41]. These cytoskeleton‐based cytoplasmic forces are transmitted to the nucleoplasm via fluctuations of the nuclear envelope [90], affecting DNA mobility, the dynamics of nuclear bodies, and the diffusion of molecules within these structures. This cascade of mechanotransduction fine‐tunes gene expression [90] and also influences mRNA splicing, as it contributes to the organization of nuclear condensates, including nuclear speckles [91]. Disruption of these forces leads to altered quantity and quality of maternal transcripts [90, 91], which in turn affect meiotic divisions [91]. These transcripts are ultimately inherited by the embryo at fertilization and may therefore impact embryonic development.

After meiosis I resumption, cytoplasmic Myosin II levels are important for maintaining oocyte ploidy. In oocytes that are too soft due to precocious chasing of Myosin II from the cortex, the excess of Myosin II in the cytoplasm and on the spindle makes chromosome capture less efficient, resulting in aneuploidy [33]. Actin filaments in mammalian oocytes also contribute to meiotic spindle formation [30], partly by regulating polar microtubule‐organizing center clustering and thus spindle pole focusing [92]. Importantly, actin filaments play a role in chromosome alignment and segregation, and their disruption leads to chromosome segregation defects and aneuploidy [29, 30, 92, 93].

### 4.2 Altered Cytoplasmic Landscape

During meiosis I, the actin cytoskeleton contributes to the organization, function and preservation of organelles in the oocyte [94, 95, 96], which is essential for proper oocyte quality and subsequent embryonic development [2, 3, 4, 97]. Actin perturbations through actin polymerization inhibiting drugs [65, 94, 98] microinjection of siRNA inhibiting the actin nucleator Formin‐like 2 [99] and conditional knockout of ArpC4 in oocytes [32] have demonstrated that an intact actin cytoskeleton is required to maintain the proper distribution of endoplasmic reticulum (ER) and mitochondria during meiosis I, ensuring asymmetric inheritance of mitochondria in favor of the oocyte, and maintaining correct fertilization‐induced Ca^2+^ oscillations essential for embryonic development. In addition, actin disruption leads to decreased mitochondrial membrane potential [32, 99] and increased ER stress [99] during meiosis I. Some of these phenotypes are observed in oocytes exhibiting increased cortical contractions due to lack of branched subcortical actin [32]. Importantly, alterations in mitochondrial positioning and function during meiosis I correlate with reduced oocyte developmental potential, including decreased success of meiosis II progression [100] and reduced fertility [32]. Moreover, reduced mitochondrial content in both human [101, 102, 103] and other mammalian oocytes [104, 105] is associated with fertilization failure and abnormal embryonic development.

### 4.3 Defects in Geometry of Division and Failed Cytokinesis

Actomyosin reorganization is essential for spindle migration during meiosis I, resulting in a highly asymmetric division in size, partly enabled by a progressive decrease in cortical tension driven by subcortical actin nucleation [31, 49]. When this nucleation is inhibited, cortical tension remains high during meiosis I, cytokinesis still occurs, but the spindle fails to migrate and the division becomes more symmetric [31]. Spindle migration during meiosis I is also impaired by inhibition of cytoplasmic actin nucleators [31, 99, 106, 107], leading to an increased proportion of mouse oocytes exhibiting symmetric divisions or abnormally large polar bodies. Preventing dismantling of the cytoplasmic actin meshwork at NEBD similarly blocks spindle migration, resulting in symmetric divisions or complete failure of division [28].

Spindle migration in meiosis I is not necessary for cytokinesis [6, 22, 23]. In contrast, formation of the actomyosin cap is essential for first polar body extrusion [72, 77, 79, 80, 108]. Failure of actin cap formation, due to impaired recruitment of the Arp2/3 complex by various means, results in cytokinesis failure, which is associated with defects in the contractile ring [72, 74, 78, 109]. However, in most oocytes lacking Formin 2, and therefore cytoplasmic actin, cortical differentiation and cortical contractions at anaphase still occur, yet cytokinesis fails, likely due to defective recruitment of Myosin II to the cleavage furrow [75]. Consistently, inhibition of Myosin II activation by different approaches [81, 82, 110] also leads to cytokinesis failure in meiosis I.

In meiosis II, disruption of the actomyosin cap similarly prevents second polar body extrusion. Loss of the cap through perturbations of Rac [66], Cdc42 [77] and Ran [44], or through Arp2/3 complex inhibition [39], leads the spindle to drift away from the cortex during metaphase II [39, 66] and anaphase II [44], thereby preventing second polar body extrusion [44, 66, 111]. Conversely, excessively strong anchoring of chromatids to the cortex following MRCKβ inhibition induces spindle distortion or breakage, resulting in either two maternal pronuclei or a maternal pronucleus enclosed within a polar‐body like structure after cytokinesis [45]. Finally, spindle rotation and cytokinesis at anaphase II onset can also be prevented in the absence of spindle midzone‐cortex interaction [43], or upon inhibition of the central spindle/RhoA pathway [44], Myosin II or actin polymerization [58], due to loss of the unilateral membrane furrow.

### 4.4 Altered Mechanics and Actomyosin Networks in Infertility Related Pathologies

Maternal age is associated with a decline in oocyte quality [11, 112, 113, 114]. Interestingly, several studies report a decrease in F‐actin levels in oocytes from aged mice, particularly in the cortical region [115, 116]. In addition, the organization of the cortical actin network itself differs in prophase I between oocytes from young and aged mice: the oocyte cortex is thicker in aged mice than in young ones, but less dense [52]. These alterations may be linked to defects in actin nucleation, as aging has been associated with degradation of actin nucleators [117], notably the Arp2/3 complex, whose cortical levels are reduced in oocytes from aged mice during meiosis I [116]. Altogether, these structural changes are likely to contribute to the disruption of the actomyosin cap in oocytes from aged mice arrested in meiosis II [117, 118], a phenotype also reported in humans [87]. Oocytes from aged mice also exhibit altered Myosin II distribution [119]. Consistently, oocyte mechanical properties are altered with maternal age [52, 120] and may contribute to their decline in quality. Specifically, oocytes from aged mice are softer in prophase I compared with those from young mice [52]. In contrast, in oocytes from aged mice arrested in metaphase II, the Young's modulus is increased [121]. In this study, cytoplasmic movement velocity (CMV), a parameter reflecting cytoplasmic dynamics and influenced by intracellular rheological properties, was also measured. CMV is decreased in oocytes from aged mice, suggesting altered cytoplasmic dynamics and global mechanical changes, potentially associated with variations in effective intracellular viscosity.

Post‐ovulatory aging, which corresponds to the period between ovulation and fertilization during which oocytes are arrested in metaphase of meiosis II, induces mechanical alterations associated with reduced oocyte quality. It is correlated with an increase in elasticity and a decrease in energy dissipation (an indirect estimate of viscosity), changes that are observed several hours before the appearance of visible morphological defects and are associated with reduced success rates of in vitro fertilization (IVF) [51]. Post‐ovulatory aging also induces dynamic, time‐dependent changes that affect both cellular stiffness and cytoplasmic movement velocity [121]. Beyond viscoelastic properties, a decrease in cortical tension, associated with altered levels and abnormal localization of Myosin II, has also been observed in oocytes subjected to post‐ovulatory aging [60]. These cortical defects are linked to reduced fertilization efficiency and increased polyspermy in IVF assays [60]. Moreover, post‐ovulatory aging has been reported to induce actin cytoskeleton disorganization, potentially altering endoplasmic reticulum and mitochondrial distribution. These structural changes may contribute to the mechanical alterations observed in post‐ovulatory aging [98]. Overall, these observations suggest a complex, heterogeneous remodeling of the cytoskeleton rather than a simple global mechanical deterioration.

Finally, some studies suggest that alterations in the mechanical properties and actomyosin networks of oocytes may be associated with specific infertility‐related pathologies. For instance, in obese mice, mitochondria activity and redox status are altered [122]. This has been partially attributed to decreased expression of the Formin INF2 [123], impairing actin polarization and resulting in mitochondrial damage and dysfunction inherited by the future embryo [124]. In contrast, other pathologies, such as premature ovarian insufficiency and polycystic ovary syndrome, have been predominantly studied at the level of the follicle and the ovary [125, 126], with limited direct evidence regarding their impact on oocyte mechanical properties.

## 5 Clinical Perspective: Mechanical Markers of Oocyte Quality in a Clinical Context

As mentioned above, assisted reproductive technologies (ART) are increasingly used, and several selection protocols are now applied to improve the chances of pregnancy. For sperm, selection is mainly based on measurable criteria such as shape, size and motility [127]. For embryos, both morphological and morphokinetic parameters are used, with more recent additions including deep learning approaches and genetic testing [128]. In contrast, oocyte selection still mainly relies on morphological criteria [129, 130], which are often more descriptive than truly predictive [131]. In this context, adding complementary markers such as mechanical properties could provide a more robust and reliable selection process.

### 5.1 Mechanical Properties and Developmental Potential, From Fundamental Research to Clinical Evidence

As discussed in the previous sections, fundamental research has demonstrated that the interplay between actomyosin networks and the mechanical properties of oocytes regulates key processes of oocyte meiotic maturation, which directly impacts their quality. From a clinical perspective, several studies have evaluated the link between oocyte mechanical properties prior to fertilization and their subsequent embryonic development. In humans, analysis of the persistence of the injection funnel after withdrawal of the intracytoplasmic sperm injection pipette suggests that lower cytoplasmic viscosity may be favorable for embryonic development [132, 133]. Moreover, the combination of multiple mechanical parameters allows prediction of blastocyst formation in humans and mice within hours after fertilization [89]. In mice, 74% of the zygotes exhibiting an intermediate stiffness and thus predicted to be viable led to a live birth, compared to 24% for zygotes too stiff or too soft and 49% for zygotes selected by morphology [19, 89]. These findings suggest that embryonic potential is largely determined by oocyte quality prior to fertilization, including its mechanical properties.

### 5.2 Techniques for Measuring Oocyte Mechanical Properties, From Experimental Tools to Clinically Compatible Approaches

**FIGURE 3:**
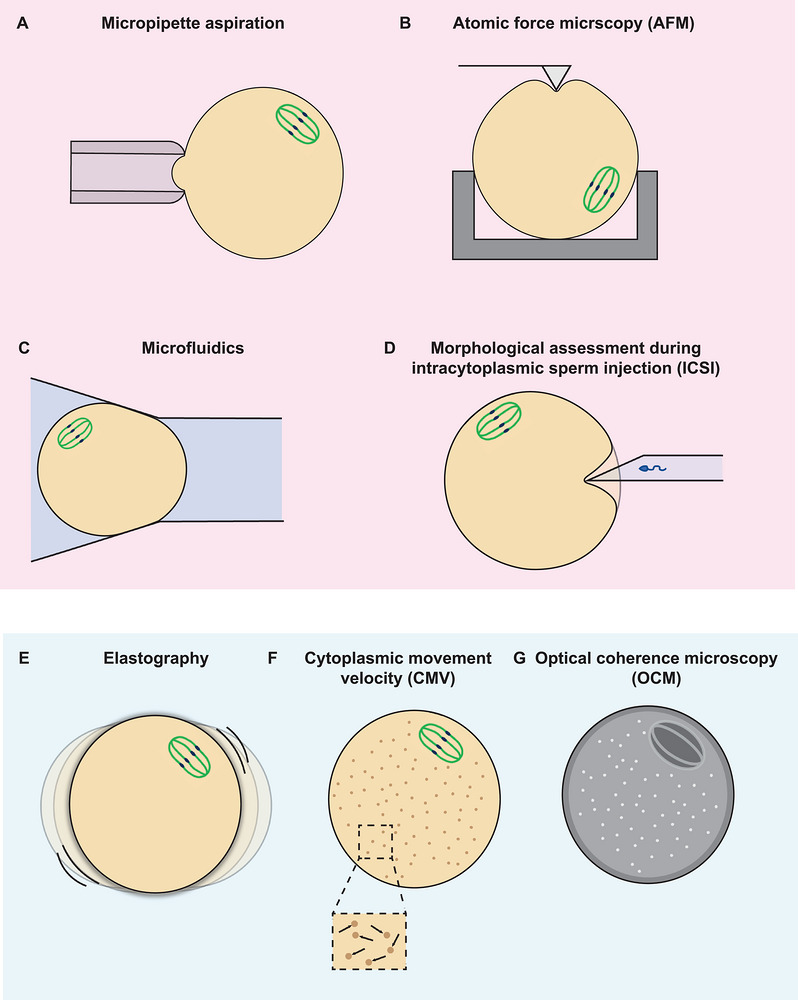
Schematic representation of the techniques used to assess oocyte mechanical properties. (A) Micropipette aspiration, with a glass capillary applying a controlled aspiration pressure to the oocyte, inducing a deformation. (B) Atomic force microscopy (AFM), with a cantilever with a tip (a triangular tip represented here) applying a controlled force to the oocyte, inducing a local indentation. (C) Microfluidics, in which the oocyte is passed through a microchannel smaller than its diameter, inducing its deformation due to confinement. (D) Intracytoplasmic sperm injection (ICSI), during which persistence and characteristics of the injection funnel formed by the sperm injection pipette (right) can be analyzed. (E) Elastography, in which high‐frequency vibrations are applied to the oocyte to map its viscoelasticity properties. (F) Cytoplasmic movement velocity (CMV), a time‐lapse imaging combined with particle image velocity (PIV) analysis to measure intracellular motion. (G) Optical coherence microscopy (OCM), a high‐resolution imaging allowing visualization of intracellular structures not accessible with standard bright‐field microscopy. The light pink box encloses techniques that induce local or global deformation to the oocyte. The light blue box encloses techniques that induce minimal or no deformation to the oocyte.

Several techniques provide robust and precise measurements of oocyte mechanical properties [134, 135, 136] (Figure 3 and Table 1). Among them, micropipette aspiration [31, 33, 49, 58, 89, 137] (Figure 3A) is relatively easy to implement as it relies on the observation of cell deformation induced by a controlled suction pressure applied via a capillary. It induces a moderate deformation of the oocyte, allowing a global measurement of cortical tension [31, 49] or visco‐elastic properties [58]. Alternative approaches such as atomic force microscopy (AFM), or micro and nano force sensing platforms allow local measurement of oocyte mechanical properties in both mouse and human [50, 62, 134] (Figure 3B). However, these methods require extensive manual micromanipulation limiting their use in routine clinical settings.

**TABLE 1:** Overview of the techniques used for assessing oocyte mechanical properties and their clinical relevance in ART. Table that summarizes the main approaches used to characterize oocyte mechanical properties, from direct deformation‐based techniques (Micropipette aspiration, Atomic force microscopy, Microfluidics, ICSI: Intracytoplasmic sperm injection) to emerging minimally or non‐invasive methods (Elastography and image‐derived approach, CMV: Cytoplasmic movement velocity and OCM: Optical coherence microscopy).

Measurement technique	Mechanical parameters assessed	Invasiveness	Clinical applicability	Relation to oocyte developmental potential	References
Micropipette aspiration	Cortical tension Viscoelastic properties	Moderate deformation (tens of micrometers)	Moderate (historical technique but not user‐friendly)	**Mouse**: Abnormal cortical tension associated with altered meiotic division geometry and aneuploidy. **Human**: viscoelastic properties predict the formation of usable blastocysts. Embryo development is not affected by the measurement. **Mouse and Human**: Correlation between intermediate zygote stiffness and zygote viability.	[31, 33, 49, 89, 152]
Atomic force microscopy	Cortical tension, elastic modulus, viscosity estimation	Local deformation (micrometers)	Limited (technically complex, costly, not user‐friendly)	**Mouse**: Lower Young's modulus of inner zona pellucida and higher viscosity associated with improved embryonic yield. Decrease stiffness with maternal age. **Human**: Increased zona pellucida Young's modulus is associated with cycles resulting in pregnancy.	[50, 51, 52]
Microfluidics	Indirect mechanical measurement through oocyte deformability	Large deformation (several tens of micrometers, flow‐induced deformation or indentation)	Promising (low‐cost, easy prototyping)	**Mouse**: Distinguish oocytes with aberrant mechanical properties but no direct correlation with oocyte developmental potential.	[62, 138, 141, 142]
Elastography	Elasticity, viscoelastic maps	Minimal deformation (sub‐micrometer, mechanically induced waves)	Emerging (minimal mechanical perturbation)	**Mouse**: Still exploratory; mechanical or dynamic readouts may reflect cellular state, but direct correlations with developmental competence remain under investigation.	[63, 145]
Image‐based approach (CMV, OCM)	Cytoplasmic dynamics; indirect mechanical readouts (cytoskeletal organization)	Non‐invasive (time‐lapse imaging)	Emerging (non‐invasive optical approach)		[121, 147, 148]
Morphological assessment during ICSI process	Indirect mechanical measurement via oocyte deformability, persistence of the injection funnel	Invasive (ISCI procedure)	High (in the frame of ART clinical practice)	**Human**: Persistent funnel associated with delayed development and reduced embryo quality.	[132, 133, 144]

Microfluidics offers a good alternative by providing integrated and automated platforms in which oocytes can undergo controlled deformation within well‐defined geometries and flow conditions [138, 139, 140]. Several studies have developed microfluidic devices based on constrictions smaller than the oocyte diameter to deform the cell and infer its mechanical properties [19, 141, 142, 143]. These approaches can effectively measure mechanical parameters, but some cause damage due to flow‐induced shear stress [141, 142]. However, a recent study has shown that it is also possible to obtain mechanical measurements without compromising oocyte integrity [62] supporting their clinical application.

Interestingly, for oocytes undergoing intracytoplasmic sperm injection (ICSI), analysis of the injection funnel persistence or the oocyte deformation during the procedure can provide indicators of the oocyte mechanical properties and, potentially, its quality [132, 133, 144] (Figure 3D). This approach has the main advantage of providing indirect mechanical information without any additional manipulation of the oocyte.

In the techniques previously described, mechanical measurement relies on the deformation of oocytes on a scale ranging from micrometers to several tens of micrometers, which subjects them to stresses that may hinder their development. In contrast, optical microelastography relies on elastic waves induced using a vibrating micropipette. Wave propagation generates submicron deformations that are tracked using ultrafast optical imaging (Figure 3E). It enables high‐resolution mapping of internal oocyte stiffness, providing localized and quantitative measurements of elasticity with acquisition times on the millisecond scale [145]. Combined with optimization‐based reconstruction techniques, optical microelastography allows the generation of viscoelastic maps of oocytes in a fully non‐invasive manner [63].

Finally, image‐based approaches may infer mechanical properties without external stimulation, relying on either cellular dynamics or structural organization. Cytoplasmic movement velocity (CMV) (Figure 3E) reflects cytoplasmic dynamics and is influenced by intracellular rheological properties and actomyosin networks [121, 146]. Similarly, optical coherence microscopy (OCM) (Figure 3F) enables visualization of intracellular structures not accessible with standard bright‐field microscopy, such as the nuclear apparatus, cytoskeletal filaments, cellular cortex, and cytoplasmic protrusions [147, 148]. Such imaging approaches could provide indirect access to cellular mechanics by analyzing structural and dynamic features, such as cytoskeletal organization or cortical architecture.

### 5.3 Integrating Mechanical Markers Into Predictive Frameworks in ART

Measurements obtained using the different techniques described above often yield multiple mechanical parameters for a single oocyte. Increasing knowledge on the mechanical properties of mammalian oocytes has prompted the development of biophysical models that seek to account for the mechanical complexity of the oocyte, often distinguishing properties of subcellular compartments [149, 150]. Rather than relying on single experimental readouts, these models can integrate data obtained through different mechanical measurement techniques [151]. This raises the question of how to interpret these multi‐parametric results to derive information useful for decision‐making regarding oocyte selection in ART processes. In particular, several studies have shown that it is necessary to combine multiple mechanical parameters to distinguish groups of oocytes that were indistinguishable using a single parameter [52, 89]. One study [152] used artificial intelligence (AI) to build a predictive classifier based on five mechanical properties measured by micropipette aspiration, in order to predict the developmental potential of human oocytes. Their machine learning‐based classifier achieved a higher predictive value than that of embryologists, highlighting the potential of integrating mechanical markers into oocyte selection frameworks. In a clinical setting, this type of AI‐based approach would be relevant, as it could reduce observer bias and variability. Interestingly, mechanical parameters could be integrated into the AI algorithms currently being evaluated in clinical settings, which rely mostly on morphological criteria [153, 154].

## 6 Future Directions

In this review we have described the pathways regulating the actomyosin networks and how they control the mechanical properties of mammalian oocytes and their proper meiotic maturation. We have presented how alterations in the actomyosin networks can result in mechanical defects, that can be linked to pathological conditions. In this context, we have presented the emerging use of mechanical properties as potential markers of quality for oocyte selection in ART. We have highlighted recent advances in the development of measurement methods and analysis algorithms aimed at integrating mechanical markers into ART procedures.

In addition to optimizing the selection of oocytes, an increasing amount of work focuses on the development of therapeutic strategies to improve both oocyte quality [155, 156, 157] and ovarian defects associated with infertility [126, 158, 159, 160]. New therapeutic strategies may also be developed to modulate mechanical properties associated with the actomyosin networks, particularly in oocytes from aged patients, in which mechanical properties have been shown to be altered [52, 120]. Strategies allowing an increase in cortical tension [119, 161] and rescue of cortical F‐actin [116] improve the ploidy of oocytes from aged mice and reduce meiotic defects. However, further studies are needed to identify the full range of molecular pathways involved in mechanical defects, in order to develop robust therapeutic strategies to improve the developmental potential of oocytes with these defects.

Importantly, procedures accompanying ART, such as in vitro maturation (IVM) or oocyte vitrification, can impact oocyte quality. Indeed, human oocytes having undergone IVM present an overall lower developmental potential [162]. Vitrified mouse oocytes have a decreased cortical tension [163] and altered actin cytoplasmic viscoelastic properties [164], potentially impacting oocyte development [163, 164]. Artificially increasing cortical tension using concanavalin A [163] or preventively dismantling the F‐actin network prior to vitrification [164] attenuate the potential deleterious effects associated with vitrification. Nonetheless, further work is necessary to understand how these procedures impact mechanical properties, in hopes of limiting their potential deleterious effects on oocyte quality.

Mechanical properties are important not only at the cellular level of the oocyte, but also within the broader context of the ovary in which it develops. Ovarian mechanical properties have been shown to impact the development of follicular structures [165, 166, 167, 168, 169] through increased ovarian stiffness associated with fibrosis due to age [170], or diseases such as polycystic ovarian syndrome and premature ovarian insufficiency [125, 171, 172]. Therapeutical strategies are being developed to address these mechanical defects in the ovary [173, 174, 175]. However, further research is needed to fully elucidate how the mechanical properties of the ovary influence those of the oocyte.

## Author Contributions

MET supervised the preparation of the review and secured funding. AS and MET conceptualized the review. AS and RB prepared the original draft. AS, RB, LB and MET reviewed and edited the manuscript.

## Conflicts of Interest

The authors declare no conflicts of interest.

## Data Availability

Data sharing not applicable to this article as no datasets were generated or analysed during the current study.

## References

[1] I. M. A. Jentoft , F. J. B. Bäuerlein , L. M. Welp , et al., “Mammalian Oocytes Store Proteins for the Early Embryo on Cytoplasmic Lattices,” Cell 186 (2023): 5308–5327.e25, 10.1016/j.cell.2023.10.003. 37922900

[2] E. Nikalayevich , N. Zollo , and M.‐H. Verlhac , “Impact of Organelle Architecture on Oocyte Developmental Potential,” Current Opinion in Cell Biology 95 (2025): 102556, 10.1016/j.ceb.2025.102556. 40505385

[3] B. Wetherall and S. Madgwick , “Peculiarities of the Mammalian Oocyte Cell Cycle,” Physiology 41, no. 4 (2026): 0, 10.1152/physiol.00027.2025. PMC7618401 41117414

[4] I. M. A. Jentoft and M. Schuh , “Protein Storage in Oocytes: Implications for Oocyte Quality, Embryonic Development, and Female Fertility,” Annual Review of Cell and Developmental Biology 41 (2025): 15–43, 10.1146/annurev-cellbio-101323-031045. 40803772

[5] F. J. Longo and D.‐Y. Chen , “Development of Cortical Polarity in Mouse Eggs: Involvement of the Meiotic Apparatus,” Developmental Biology 107 (1985): 382–394, 10.1016/0012-1606(85)90320-3. 4038667

[6] M. H. Verlhac , C. Lefebvre , P. Guillaud , P. Rassinier , and B. Maro , “Asymmetric Division in Mouse Oocytes: with or without Mos,” Current biology 10 (2000): 1303–1306, 10.1016/S0960-9822(00)00753-3. 11069114

[7] Z. Holubcova , M. Blayney , K. Elder , and M. Schuh , “Error‐Prone Chromosome‐Mediated Spindle Assembly Favors Chromosome Segregation Defects in Human Oocytes,” Science 348 (2015): 1143–1147, 10.1126/science.aaa9529. 26045437 PMC4477045

[8] H. Kyogoku and T. S. Kitajima , “Large Cytoplasm Is Linked to the Error‐Prone Nature of Oocytes,” Developmental Cell 41 (2017): 287–298.e4, 10.1016/j.devcel.2017.04.009. 28486131

[9] D. Cimadomo , G. Fabozzi , A. Vaiarelli , N. Ubaldi , F. M. Ubaldi , and L. Rienzi , “Impact of Maternal Age on Oocyte and Embryo Competence,” Frontiers in Endocrinology 9 (2018): 327, 10.3389/fendo.2018.00327. 30008696 PMC6033961

[10] R. Gruhn , A. P. Zielinska , V. Shukla , et al., “Chromosome Errors in Human Eggs Shape Natural Fertility over Reproductive Life Span,” Science 365 (2019): 1466–1469, 10.1126/science.aav7321. 31604276 PMC7212007

[11] D. Bebbere , G. Coticchio , A. Borini , and S. Ledda , “Oocyte Aging: Looking beyond Chromosome Segregation Errors,” Journal of Assisted Reproduction and Genetics 39 (2022): 793–800, 10.1007/s10815-022-02441-z. 35212880 PMC9051005

[12] C. Charalambous , A. Webster , and M. Schuh , “Aneuploidy in Mammalian Oocytes and the Impact of Maternal Ageing,” Nature Reviews Molecular Cell Biology 24 (2023): 27–44, 10.1038/s41580-022-00517-3. 36068367

[13] S. Galatidou , A. Peteleski , L. Mchuggh , et al., “O‐033 Molecular Insights into the Decline in Oocyte Quality with Advanced Maternal Age: Findings from Comprehensive Single Cell‐Omics Analysis of 112 Human Oocytes,” Human Reproduction 39 (2024): deae108033.

[14] C. Voros , F. Chatzinikolaou , G. Papadimas , et al., “Translational Fidelity Decline in the Aging Oocyte and Embryo Development,” International Journal of Molecular Sciences 27 (2026): 2614, 10.3390/ijms27062614. 41898476 PMC13027342

[15] D. Szollosi , P. Calarco , and R. P. Donahue , “Absence of Centrioles in the First and Second Meiotic Spindles of Mouse Oocytes,” Journal of Cell Science 11 (1972): 521–541, 10.1242/jcs.11.2.521. 5076360

[16] G. Manandhar , H. Schatten , and P. Sutovsky , “Centrosome Reduction During Gametogenesis and Its Significance1,” Biology of Reproduction 72 (2005): 2–13, 10.1095/biolreprod.104.031245. 15385423

[17] D. M. Ely and B. E. Hamilton , “Trends in Fertility and Mother's Age at First Birth Among Rural and Metropolitan Counties: United States, 2007‐2017,” NCHS Data Brief 323 (2018): 1–8. 30475685

[18] D. K. Gardner and B. Balaban , “Assessment of Human Embryo Development Using Morphological Criteria in an Era of Time‐Lapse, Algorithms and ‘OMICS’: Is Looking Good Still Important?,” Molecular Human Reproduction 22 (2016): 704–718, 10.1093/molehr/gaw057. 27578774

[19] L. Z. Yanez and D. B. Camarillo , “Microfluidic Analysis of Oocyte and Embryo Biomechanical Properties to Improve Outcomes in Assisted Reproductive Technologies,” MHR: Basic Science of Reproductive Medicine 23 (2017): 235–247, 10.1093/molehr/gaw071. 27932552 PMC5909856

[20] M. Fluks , R. Collier , A. Walewska , A. W. Bruce , and A. Ajduk , “How Great Thou ART: Biomechanical Properties of Oocytes and Embryos as Indicators of Quality in Assisted Reproductive Technologies,” Frontiers in Cell and Developmental Biology 12 (2024): 1342905. 38425501 10.3389/fcell.2024.1342905 PMC10902081

[21] M. Murrell , P. W. Oakes , M. Lenz , and M. L. Gardel , “Forcing Cells into Shape: the Mechanics of Actomyosin Contractility,” Nature Reviews Molecular Cell Biology 16 (2015): 486–498, 10.1038/nrm4012. 26130009 PMC7443980

[22] B. Leader , H. Lim , M. J. Carabatsos , et al., “Formin‐2, Polyploidy, Hypofertility and Positioning of the Meiotic Spindle in Mouse Oocytes,” Nature Cell Biology 4 (2002): 921–928, 10.1038/ncb880. 12447394

[23] J. Dumont , K. Million , K. Sunderland , et al., “Formin‐2 Is Required for Spindle Migration and for the Late Steps of Cytokinesis in Mouse Oocytes,” Developmental Biology 301 (2007): 254–265, 10.1016/j.ydbio.2006.08.044. 16989804

[24] J. Azoury , K. W. Lee , V. Georget , P. Rassinier , B. Leader , and M.‐H. Verlhac , “Spindle Positioning in Mouse Oocytes Relies on a Dynamic Meshwork of Actin Filaments,” Current Biology 18 (2008): 1514–1519, 10.1016/j.cub.2008.08.044. 18848445

[25] M. Schuh and J. Ellenberg , “A New Model for Asymmetric Spindle Positioning in Mouse Oocytes,” Current Biology 18 (2008): 1986–1992, 10.1016/j.cub.2008.11.022. 19062278

[26] S. Pfender , V. Kuznetsov , S. Pleiser , E. Kerkhoff , and M. Schuh , “Spire‐Type Actin Nucleators Cooperate with Formin‐2 to Drive Asymmetric Oocyte Division,” Current Biology 21 (2011): 955–960, 10.1016/j.cub.2011.04.029. 21620703 PMC3128265

[27] P. Montaville , A. Jégou , J. Pernier , et al., “Spire and Formin 2 Synergize and Antagonize in Regulating Actin Assembly in Meiosis by a Ping‐Pong Mechanism,” PLoS Biology 12 (2014): 1001795, 10.1371/journal.pbio.1001795. PMC3934834 24586110

[28] J. Azoury , K. W. Lee , V. Georget , P. Hikal , and M.‐H. Verlhac , “Symmetry Breaking in Mouse Oocytes Requires Transient F‐actin Meshwork Destabilization,” Development 138 (2011): 2903–2908, 10.1242/dev.060269. 21653611

[29] B. Mogessie and M. Schuh , “Actin Protects Mammalian Eggs against Chromosome Segregation Errors,” Science 357 (2017): aal1647. 10.1126/science.aal1647 28839045

[30] J. Roeles and G. Tsiavaliaris , “Actin‐Microtubule Interplay Coordinates Spindle Assembly in Human Oocytes,” Nature Communications 10 (2019): 4651, 10.1038/s41467-019-12674-9. PMC6789129 31604948

[31] A. Chaigne , C. Campillo , N. S. Gov , et al., “A Soft Cortex Is Essential for Asymmetric Spindle Positioning in Mouse Oocytes,” Nature Cell Biology 15 (2013): 958–966, 10.1038/ncb2799. 23851486

[32] E. Nikalayevich , G. Letort , G. de Labbey , et al., “Aberrant Cortex Contractions Impact Mammalian Oocyte Quality,” Developmental Cell 59 (2024): 841–852.e7, 10.1016/j.devcel.2024.01.027. 38387459

[33] I. Bennabi , F. Crozet , E. Nikalayevich , et al., “Artificially Decreasing Cortical Tension Generates Aneuploidy in Mouse Oocytes,” Nature Communications 11 (2020): 1649, 10.1038/s41467-020-15470-y. PMC7125192 32245998

[34] B. A. Truong Quang , R. Peters , D. A. D. Cassani , et al., “Extent of Myosin Penetration within the Actin Cortex Regulates Cell Surface Mechanics,” Nature Communications 12 (2021): 6511, 10.1038/s41467-021-26611-2. PMC8586027 34764258

[35] B. Maro , M. H. Johnson , S. J. Pickering , and G. Flach , “Changes in Actin Distribution during Fertilization of the Mouse Egg,” Journal of Embryology and Experimental Morphology 81 (1984): 211–237. 6540795

[36] J. Van Blerkom and H. Bell , “Regulation of Development in the Fully Grown Mouse Oocyte: Chromosome‐Mediated Temporal and Spatial Differentiation of the Cytoplasm and Plasma Membrane,” Journal of Embryology and Experimental Morphology 93 (1986): 213–238. 3734683

[37] Z.‐Y. Zhu , D.‐Y. Chen , J.‐S. Li , et al., “Rotation of Meiotic Spindle Is Controlled by Microfilaments in Mouse Oocytes1,” Biology of Reproduction 68 (2003): 943–946, 10.1095/biolreprod.102.009910. 12604646

[38] Q. Wang , C. Racowsky , and M. Deng , “Mechanism of the Chromosome‐Induced Polar Body Extrusion in Mouse Eggs,” Cell Division 6 (2011): 17, 10.1186/1747-1028-6-17. 21867530 PMC3179692

[39] K. Yi , J. R. Unruh , M. Deng , B. D. Slaughter , B. Rubinstein , and R. Li , “Dynamic Maintenance of Asymmetric Meiotic Spindle Position through Arp2/3‐Complex‐Driven Cytoplasmic Streaming in Mouse Oocytes,” Nature Cell Biology 13 (2011): 1252–1258, 10.1038/ncb2320. 21874009 PMC3523671

[40] K. Yi , B. Rubinstein , J. R. Unruh , F. Guo , B. D. Slaughter , and R. Li , “Sequential Actin‐Based Pushing Forces Drive Meiosis I Chromosome Migration and Symmetry Breaking in Oocytes,” Journal of Cell Biology 200 (2013): 567–576, 10.1083/jcb.201211068. 23439682 PMC3587830

[41] M. Almonacid , W. W. Ahmed , M. Bussonnier , et al., “Active Diffusion Positions the Nucleus in Mouse Oocytes,” Nature Cell Biology 17 (2015): 470–479, 10.1038/ncb3131. 25774831

[42] A. Colin , G. Letort , N. Razin , et al., “Active Diffusion in Oocytes Nonspecifically Centers Large Objects during Prophase I and Meiosis I,” Journal of Cell Biology 219 (2020): 201908195, 10.1083/jcb.201908195. PMC7054987 31952078

[43] H. Wang , Y. Li , J. Yang , et al., “Symmetry Breaking in Hydrodynamic Forces Drives Meiotic Spindle Rotation in Mammalian Oocytes,” Science Advances 6 (2020): aaz5004, 10.1126/sciadv.aaz5004. PMC7124937 32284983

[44] B. Dehapiot , R. Clément , A. Bourdais , V. Carrière , S. Huet , and G. Halet , “RhoA‐and Cdc42‐Induced Antagonistic Forces Underlie Symmetry Breaking and Spindle Rotation in Mouse Oocytes,” PLoS Biology 19 (2021): 3001376. 10.1371/journal.pbio.3001376 PMC8448345 34491981

[45] A. Bourdais , B. Dehapiot , and G. Halet , “MRCK Activates Mouse Oocyte Myosin II for Spindle Rotation and Male Pronucleus Centration,” Journal of Cell Biology 222 (2023): 202211029. 10.1083/jcb.202211029 PMC10470461 37651121

[46] Y. Hao , S. Cheng , Y. Tanaka , Y. Hosokawa , Y. Yalikun , and M. Li , “Mechanical Properties of Single Cells: Measurement Methods and Applications,” Biotechnology Advances 45 (2020): 107648, 10.1016/j.biotechadv.2020.107648. 33080313

[47] H. Schillers , M. Wälte , K. Urbanova , and H. Oberleithner , “Real‐Time Monitoring of Cell Elasticity Reveals Oscillating Myosin Activity,” Biophysical Journal 99 (2010): 3639–3646, 10.1016/j.bpj.2010.09.048. 21112288 PMC2998603

[48] E. Moeendarbary and A. R. Harris , “Cell Mechanics: Principles, Practices, and Prospects,” Wiley Interdisciplinary Reviews: Systems Biology and Medicine 6 (2014): 371–388. 25269160 10.1002/wsbm.1275 PMC4309479

[49] A. Chaigne , C. Campillo , N. S. Gov , et al., “A Narrow Window of Cortical Tension Guides Asymmetric Spindle Positioning in the Mouse Oocyte,” Nature Communications 6 (2015): 6027, 10.1038/ncomms7027. 25597399

[50] L. Andolfi , E. Masiero , E. Giolo , et al., “Investigating the Mechanical Properties of Zona Pellucida of Whole Human Oocytes by Atomic Force Spectroscopy,” Integrative Biology 8 (2016): 886–893, 10.1039/c6ib00044d. 27476747

[51] A. Battistella , L. Andolfi , and M. Zanetti , “Atomic Force Spectroscopy‐Based Essay to Evaluate Oocyte Postovulatory Aging,” Bioengineering & Translational Medicine 7 (2022): 10294. 10.1002/btm2.10294 PMC9472013 36176606

[52] R. Bulteau , L. Barbier , G. Lamour , et al., “Atomic Force Microscopy Reveals Differences in Mechanical Properties Linked to Cortical Structure in Mouse and Human Oocytes,” Small 21 (2025): 2500221. 40159757 10.1002/smll.202500221 PMC12288798

[53] Y. Du , Y. Cai , Z. Yang , K. Gao , M. Sun , and X. Zhao , “Modeling and Validation of Oocyte Mechanical Behavior Using AFM Measurement and Multiphysics Simulation,” Sensors 25 (2025): 5479, 10.3390/s25175479. 40942919 PMC12431014

[54] J.‐Y. Tinevez , U. Schulze , G. Salbreux , J. Roensch , J.‐F. Joanny , and E. Paluch , “Role of Cortical Tension in Bleb Growth,” Proceedings of the National Academy of Sciences 106 (2009): 18581–18586, 10.1073/pnas.0903353106. PMC2765453 19846787

[55] G. Salbreux , G. Charras , and E. Paluch , “Actin Cortex Mechanics and Cellular Morphogenesis,” Trends in Cell Biology 22 (2012): 536–545, 10.1016/j.tcb.2012.07.001. 22871642

[56] E. Fischer‐Friedrich , Y. Toyoda , C. J. Cattin , D. J. Müller , A. A. Hyman , and F. Jülicher , “Rheology of the Active Cell Cortex in Mitosis,” Biophysical Journal 111 (2016): 589–600, 10.1016/j.bpj.2016.06.008. 27508442 PMC4982928

[57] P. Chugh , A. G. Clark , M. B. Smith , et al., “Actin Cortex Architecture Regulates Cell Surface Tension,” Nature Cell Biology 19 (2017): 689–697, 10.1038/ncb3525. 28530659 PMC5536221

[58] S. M. Larson , H. J. Lee , P. Hung , L. M. Matthews , D. N. Robinson , and J. P. Evans , “Cortical Mechanics and Meiosis II Completion in Mammalian Oocytes Are Mediated by Myosin‐II and Ezrin‐Radixin‐Moesin (ERM) Proteins,” Molecular Biology of the Cell 21 (2010): 3182–3192, 10.1091/mbc.e10-01-0066. 20660156 PMC2938384

[59] A. Jégou , F. Pincet , E. Perez , J. P. Wolf , A. Ziyyat , and C. Gourier , “Mapping Mouse Gamete Interaction Forces Reveal Several Oocyte Membrane Regions with Different Mechanical and Adhesive Properties,” Langmuir 24 (2008): 1451–1458. 18027975 10.1021/la702258x

[60] A. C. L. Mackenzie , D. D. Kyle , L. A. McGinnis , et al., “Cortical Mechanics and Myosin‐II Abnormalities Associated with Post‐Ovulatory Aging: Implications for Functional Defects in Aged Eggs,” Molecular Human Reproduction 22 (2016): 397–409, 10.1093/molehr/gaw019. 26921397 PMC4884917

[61] B. E. Vos , Y. Vadapalli , T. Muenker , et al., “Direct Mechanical Communication of Cellular to Nuclear Shapes in Oocytes,” Biophysical Journal 125 (2025): 2978–2990.40831060 10.1016/j.bpj.2025.08.012

[62] L. Barbier , R. Bulteau , B. Rezaei , et al., “Noninvasive Characterization of Oocyte Deformability in Microconstrictions,” Science Advances 11 (2025): adr9869, 10.1126/sciadv.adr9869. PMC11838009 39970229

[63] G. Flé , E. Van Houten , G. Rémillard‐Labrosse , G. FitzHarris , and G. Cloutier , “Imaging the Subcellular Viscoelastic Properties of Mouse Oocytes,” Proceedings of the National Academy of Sciences 120 (2023): 2213836120. 10.1073/pnas.2213836120 PMC10214128 37186851

[64] I. Krause , U. Pohler , S. Grosse , et al., “Characterization of the Injection Funnel during Intracytoplasmic Sperm Injection Reflects Cytoplasmic Maturity of the Oocyte,” Fertility and Sterility 106 (2016): 1101–1106, 10.1016/j.fertnstert.2016.06.015. 27336210

[65] G. FitzHarris , P. Marangos , and J. Carroll , “Changes in Endoplasmic Reticulum Structure during Mouse Oocyte Maturation Are Controlled by the Cytoskeleton and Cytoplasmic Dynein,” Developmental Biology 305 (2007): 133–144, 10.1016/j.ydbio.2007.02.006. 17368610

[66] G. Halet and J. Carroll , “Rac Activity Is Polarized and Regulates Meiotic Spindle Stability and Anchoring in Mammalian Oocytes,” Developmental Cell 12 (2007): 309–317, 10.1016/j.devcel.2006.12.010. 17276347

[67] C. Simerly , G. Nowak , P. de Lanerolle , and G. Schatten , “Differential Expression and Functions of Cortical Myosin IIA and IIB Isotypes during Meiotic Maturation, Fertilization, and Mitosis in Mouse Oocytes and Embryos,” Molecular Biology of the Cell 9 (1998): 2509–2525, 10.1091/mbc.9.9.2509. 9725909 PMC25518

[68] N. Liu , R. Kawamura , W. Qiang , et al., “Isolation and Manipulation of Meiotic Spindles from Mouse Oocytes Reveals Migration Regulated by Pulling Force during Asymmetric Division,” bioRxiv, preprint (2025): 2024–3012, 10.1101/2024.12.06.627260.

[69] H. Li , F. Guo , B. Rubinstein , and R. Li , “Actin‐Driven Chromosomal Motility Leads to Symmetry Breaking in Mammalian Meiotic Oocytes,” Nature Cell Biology 10 (2008): 1301–1308, 10.1038/ncb1788. 18836438

[70] K. Yi , B. Rubinstein , and R. Li , “Symmetry Breaking and Polarity Establishment during Mouse Oocyte Maturation,” Philosophical Transactions of the Royal Society B: Biological Sciences 368 (2013): 20130002, 10.1098/rstb.2013.0002. PMC3785956 24062576

[71] X. Duan , Y. Li , K. Yi , et al., “Dynamic Organelle Distribution Initiates Actin‐Based Spindle Migration in Mouse Oocytes,” Nature Communications 11 (2020): 277, 10.1038/s41467-019-14068-3. PMC6959240 31937754

[72] Z.‐B. Wang , Z.‐Z. Jiang , Q.‐H. Zhang , et al., “Specific Deletion of Cdc42 Does Not Affect Meiotic Spindle Organization/Migration and Homologous Chromosome Segregation but Disrupts Polarity Establishment and Cytokinesis in Mouse Oocytes,” Molecular Biology of the Cell 24 (2013): 3832–3841, 10.1091/mbc.e13-03-0123. 24131996 PMC3861080

[73] M. Deng , P. Suraneni , R. M. Schultz , and R. Li , “The Ran GTPase Mediates Chromatin Signaling to Control Cortical Polarity during Polar Body Extrusion in Mouse Oocytes,” Developmental Cell 12 (2007): 301–308, 10.1016/j.devcel.2006.11.008. 17276346

[74] S.‐C. Sun , Z.‐B. Wang , Y.‐N. Xu , S.‐E. Lee , X.‐S. Cui , and N.‐H. Kim , “Arp2/3 Complex Regulates Asymmetric Division and Cytokinesis in Mouse Oocytes,” PLoS ONE 6 (2011): 18392, 10.1371/journal.pone.0018392. PMC3072972 21494665

[75] J. Dumont , S. Petri , F. Pellegrin , et al., “A Centriole‐ and RanGTP‐Independent Spindle Assembly Pathway in Meiosis I of Vertebrate Oocytes,” The Journal of Cell Biology 176 (2007): 295–305, 10.1083/jcb.200605199. 17261848 PMC2063956

[76] W. S. Yuen , Q. H. Zhang , A. Bourdais , D. Adhikari , G. Halet , and J. Carroll , “Polo‐Like Kinase 1 Promotes Cdc42‐Induced Actin Polymerization for Asymmetric Division in Oocytes,” Open Biology 13 (2023): 220326, 10.1098/rsob.220326. 36883283 PMC9993042

[77] B. Dehapiot , V. Carrière , J. Carroll , and G. Halet , “Polarized Cdc42 Activation Promotes Polar Body Protrusion and Asymmetric Division in Mouse Oocytes,” Developmental Biology 377 (2013): 202–212, 10.1016/j.ydbio.2013.01.029. 23384564 PMC3690527

[78] Q.‐C. Wang , X. Wan , R.‐X. Jia , et al., “Inhibition of N‐WASP Affects Actin‐Mediated Cytokinesis during Porcine Oocyte Maturation,” Theriogenology 144 (2020): 132–138, 10.1016/j.theriogenology.2020.01.005. 31940504

[79] J. Elbaz , Y. Reizel , N. Nevo , D. Galiani , and N. Dekel , “Epithelial Cell Transforming Protein 2 (ECT2) Depletion Blocks Polar Body Extrusion and Generates Mouse Oocytes Containing Two Metaphase II Spindles,” Endocrinology 151 (2010): 755–765, 10.1210/en.2009-0830. 19996184

[80] Y. Zhang , X. Duan , R. Cao , et al., “Small GTPase RhoA Regulates Cytoskeleton Dynamics during Porcine Oocyte Maturation and Early Embryo Development,” Cell Cycle 13 (2014): 3390–3403, 10.4161/15384101.2014.952967. 25485583 PMC4613651

[81] S.‐R. Lee , Y.‐N. Xu , Y.‐J. Jo , S. Namgoong , and N.‐H. Kim , “The Rho‐GTPase Effector ROCK Regulates Meiotic Maturation of the Bovine Oocyte via Myosin Light Chain Phosphorylation and Cofilin Phosphorylation,” Molecular Reproduction and Development 82 (2015): 849–858, 10.1002/mrd.22524. 26175189

[82] X. Duan , J. Liu , C.‐C. Zhu , et al., “RhoA‐Mediated MLC2 Regulates Actin Dynamics for Cytokinesis in Meiosis,” Cell Cycle 15 (2015): 471–477, 10.1080/15384101.2015.1128590. 26701676 PMC4943703

[83] Z. Wei , J. Greaney , C. Zhou , and H. A. Homer , “Cdk1 Inactivation Induces Post‐Anaphase‐Onset Spindle Migration and Membrane Protrusion Required for Extreme Asymmetry in Mouse Oocytes,” Nature Communications 9 (2018): 4029, 10.1038/s41467-018-06510-9. PMC6168559 30279413

[84] M. Glotzer , “The Molecular Requirements for Cytokinesis,” Science 307 (2005): 1735–1739, 10.1126/science.1096896. 15774750

[85] H. S. Ko , J. S. Kim , S. M. Cho , et al., “Urokinase‐Type Plasminogen Activator Expression and Rac1/WAVE‐2/Arp2/3 Pathway Are Blocked by Pterostilbene to Suppress Cell Migration and Invasion in MDA‐MB‐231 Cells,” Bioorganic & Medicinal Chemistry Letters 24 (2014): 1176–1179, 10.1016/j.bmcl.2013.12.115. 24440300

[86] T. Zhou , C.‐H. Wang , H. Yan , et al., “Inhibition of the Rac1‐WAVE2‐Arp2/3 Signaling Pathway Promotes Radiosensitivity via Downregulation of Cofilin‐1 in U251 Human Glioma Cells,” Molecular Medicine Reports 13 (2016): 4414–4420, 10.3892/mmr.2016.5088. 27052944

[87] G. Coticchio , M. C. Guglielmo , D. F. Albertini , et al., “Contributions of the Actin Cytoskeleton to the Emergence of Polarity during Maturation in Human Oocytes,” MHR: Basic Science of Reproductive Medicine 20 (2014): 200–207, 10.1093/molehr/gat085. 24258450

[88] Z.‐S. Zhong , L.‐J. Huo , C.‐G. Liang , D.‐Y. Chen , and Q.‐Y. Sun , “Small GTPase RhoA Is Required for Ooplasmic Segregation and Spindle Rotation, but Not for Spindle Organization and Chromosome Separation during Mouse Oocyte Maturation, Fertilization, and Early Cleavage,” Molecular Reproduction and Development 71 (2005): 256–261, 10.1002/mrd.20253. 15791586

[89] L. Z. Yanez , J. Han , B. B. Behr , R. A. R. Pera , and D. B. Camarillo , “Human Oocyte Developmental Potential Is Predicted by Mechanical Properties within Hours after Fertilization,” Nature Communications 7 (2016): 10809, 10.1038/ncomms10809. PMC4770082 26904963

[90] M. Almonacid , A. Al Jord , S. El‐Hayek , et al., “Active Fluctuations of the Nuclear Envelope Shape the Transcriptional Dynamics in Oocytes,” Developmental Cell 51 (2019): 145–157.e10, 10.1016/j.devcel.2019.09.010. 31607652

[91] A. Al Jord , G. Letort , S. Chanet , et al., “Cytoplasmic Forces Functionally Reorganize Nuclear Condensates in Oocytes,” Nature Communications 13 (2022): 5070. 10.1038/s41467-022-32675-5 PMC9424315 36038550

[92] E. J. Soto‐Moreno , N. N. Ali , F. Küllmer , et al., “Spindle‐Localized F‐Actin Regulates Polar MTOC Organization and the Fidelity of Meiotic Spindle Formation,” Nature Communications 16 (2025): 8323, 10.1038/s41467-025-63586-w. PMC12449456 40973727

[93] S. Dunkley and B. Mogessie , “Actin Limits Egg Aneuploidies Associated with Female Reproductive Aging,” Science Advances 9 (2023): adc9161, 10.1126/sciadv.adc9161. PMC9858517 36662854

[94] C. M. Dalton and J. Carroll , “Biased Inheritance of Mitochondria during Asymmetric Cell Division in the Mouse Oocyte,” Journal of Cell Science 126 (2013): 2955–2964. 23659999 10.1242/jcs.128744 PMC3699109

[95] I.‐W. Lee , D. Adhikari , and J. Carroll , “Miro1 Depletion Disrupts Spatial Distribution of Mitochondria and Leads to Oocyte Maturation Defects,” Frontiers in Cell and Developmental Biology 10 (2022): 986454, 10.3389/fcell.2022.986454. 36325364 PMC9619047

[96] D. Bahety , E. Böke , and A. Rodríguez‐Nuevo , “Mitochondrial Morphology, Distribution and Activity during Oocyte Development,” Trends in Endocrinology & Metabolism 35 (2024): 902–917, 10.1016/j.tem.2024.03.002. 38599901

[97] I.‐W. Lee , A. P. Tazehkand , Z.‐Y. Sha , D. Adhikari , and J. Carroll , “An Aggregated Mitochondrial Distribution in Preimplantation Embryos Disrupts Nuclear Morphology, Function, and Developmental Potential,” Proceedings of the National Academy of Sciences 121 (2024): 2317316121, 10.1073/pnas.2317316121. PMC11228517 38917013

[98] M. Szpila , A. Walewska , D. Sabat‐Pospiech , et al., “Postovulatory Ageing Modifies Sperm‐Induced Ca^2+^ Oscillations in Mouse Oocytes through a Conditions‐Dependent, Multi‐Pathway Mechanism,” Scientific Reports 9 (2019): 11859, 10.1038/s41598-019-48281-3. 31413272 PMC6694115

[99] M.‐H. Pan , K.‐H. Zhang , S.‐L. Wu , et al., “FMNL2 Regulates Actin for Endoplasmic Reticulum and Mitochondria Distribution in Oocyte Meiosis,” Elife 12 (2024): RP92732, 10.7554/eLife.92732.3. 38747713 PMC11095938

[100] Y. A. Ryabukha , O. V. Zatsepina , and Y. P. Rubtsov , “The Completing of the Second Meiotic Division by MII Mouse Oocytes Correlates with the Positioning of F‐Actin and Mitochondria in the Ooplasm,” Biochimie 230 (2025): 55–67, 10.1016/j.biochi.2024.11.004. 39577618

[101] P. Reynier , P. May‐Panloup , M.‐F. Chretien , et al., “Mitochondrial DNA Content Affects the Fertilizability of Human Oocytes,” Molecular Human Reproduction 7 (2001): 425–429, 10.1093/molehr/7.5.425. 11331664

[102] T. A. Santos , S. El Shourbagy , and J. C. S. John , “Mitochondrial Content Reflects Oocyte Variability and Fertilization Outcome,” Fertility and Sterility 85 (2006): 584–591, 10.1016/j.fertnstert.2005.09.017. 16500323

[103] Y. Murakoshi , K. Sueoka , K. Takahashi , et al., “Embryo Developmental Capability and Pregnancy Outcome Are Related to the Mitochondrial DNA Copy Number and Ooplasmic Volume,” Journal of Assisted Reproduction and Genetics 30 (2013): 1367–1375, 10.1007/s10815-013-0062-6. 23897005 PMC3824848

[104] H. Ge , T. L. Tollner , Z. Hu , et al., “The Importance of Mitochondrial Metabolic Activity and Mitochondrial DNA Replication during Oocyte Maturation in Vitro on Oocyte Quality and Subsequent Embryo Developmental Competence,” Molecular Reproduction and Development 79 (2012): 392–401, 10.1002/mrd.22042. 22467220

[105] S. H. El Shourbagy , E. C. Spikings , M. Freitas , and J. C. St John , “Mitochondria Directly Influence Fertilisation Outcome in the Pig,” Reproduction 131 (2006): 233–245. 16452717 10.1530/rep.1.00551

[106] B. Dehapiot and G. Halet , “Ran GTPase Promotes Oocyte Polarization by Regulating ERM (Ezrin/Radixin/Moesin) Inactivation,” Cell Cycle 12 (2013): 1672–1678, 10.4161/cc.24901. 23656777 PMC3713125

[107] W.‐I. Jang , Y.‐J. Jo , H.‐C. Kim , J.‐L. Jia , S. Namgoong , and N.‐H. Kim , “Non‐Muscle Tropomyosin (Tpm3) Is Crucial for Asymmetric Cell Division and Maintenance of Cortical Integrity in Mouse Oocytes,” Cell Cycle 13 (2014): 2359–2369, 10.4161/cc.29333. 25483187 PMC4128881

[108] Y. Zhang , X. Duan , B. Xiong , et al., “ROCK Inhibitor Y‐27632 Prevents Porcine Oocyte Maturation,” Theriogenology 82 (2014): 49–56, 10.1016/j.theriogenology.2014.02.020. 24681214

[109] S.‐C. Sun , Q.‐Y. Sun , and N.‐H. Kim , “JMY Is Required for Asymmetric Division and Cytokinesis in Mouse Oocytes,” Molecular Human Reproduction 17 (2011): 296–304, 10.1093/molehr/gar006. 21266449

[110] H. Suzuki , K. Koyama , K. Kabashima , J. Fang , and M. Matsuzaki , “Temporary Inhibition of Germinal Vesicle Breakdown by Rho Kinase Inhibitor Y‐27632 Is Detrimental to Oocyte Maturation,” Journal of Mammalian Ova Research 28 (2011): 126–130, 10.1274/jmor.28.126.

[111] S.‐J. Song , Q.‐C. Wang , R.‐X. Jia , X.‐S. Cui , N.‐H. Kim , and S.‐C. Sun , “Inhibition of Rac1 GTPase Activity Affects Porcine Oocyte Maturation and Early Embryo Development,” Scientific Reports 6 (2016): 34415, 10.1038/srep34415. 27694954 PMC5046063

[112] S. I. Nagaoka , T. J. Hassold , and P. A. Hunt , “Human Aneuploidy: Mechanisms and New Insights into an Age‐Old Problem,” Nature Reviews Genetics 13 (2012): 493–504, 10.1038/nrg3245. PMC3551553 22705668

[113] A. I. Mihajlović , J. Haverfield , and G. FitzHarris , “Distinct Classes of Lagging Chromosome Underpin Age‐Related Oocyte Aneuploidy in Mouse,” Developmental Cell 56 (2021): 2273–2283.e3, 10.1016/j.devcel.2021.07.022. 34428397

[114] E. A. Gaylord , M. H. Foecke , R. M. Samuel , et al., “Comparative Analysis of Human and Mouse Ovaries across Age,” Science 390 (2025): adx0659, 10.1126/science.adx0659. 41066539

[115] X. Liu , R. Fernandes , A. Jurisicova , R. F. Casper , and Y. Sun , “In Situ Mechanical Characterization of Mouse Oocytes Using a Cell Holding Device,” Lab on a Chip 10 (2010): 2154–2161, 10.1039/c004706f. 20544113

[116] C. Liu , H. Zhang , J. Mao , et al., “Mevalonate Metabolites Boost Aged Oocyte Quality through Prenylation of Small GTPases,” Nature Aging 5 (2025): 2022–2038, 10.1038/s43587-025-00946-7. 40858817 PMC12532580

[117] S.‐C. Sun , W.‐W. Gao , Y.‐N. Xu , et al., “Degradation of Actin Nucleators Affects Cortical Polarity of Aged Mouse Oocytes,” Fertility and Sterility 97 (2012): 984–990, 10.1016/j.fertnstert.2012.01.101. 22306711

[118] M. Webb , S. K. Howlett , and B. Maro , “Parthenogenesis and Cytoskeletal Organization in Ageing Mouse Eggs,” Journal of Embryology and Experimental Morphology 95 (1986): 131–145. 3794588

[119] Q. Zhuan , J. Li , G. Zhou , et al., “Procyanidin B2 Protects Aged Oocytes against Meiotic Defects through Cortical Tension Modulation,” Frontiers in Veterinary Science 9 (2022): 795050, 10.3389/fvets.2022.795050. 35464357 PMC9024290

[120] X. Liu , J. Shi , Z. Zong , K.‐T. Wan , and Y. Sun , “Elastic and Viscoelastic Characterization of Mouse Oocytes Using Micropipette Indentation,” Annals of Biomedical Engineering 40 (2012): 2122–2130, 10.1007/s10439-012-0595-3. 22644532

[121] M. Fluks , A. Rak , and A. Ajduk , “Cytoplasmic Movement Velocity in Unfertilized Mouse Oocytes: a Supportive but Not Definitive Marker of Embryo Quality,” Theriogenology 251 (2026): 117725, 10.1016/j.theriogenology.2025.117725. 41172594

[122] N. Igosheva , A. Y. Abramov , L. Poston , et al., “Maternal Diet‐Induced Obesity Alters Mitochondrial Activity and Redox Status in Mouse Oocytes and Zygotes,” PLoS ONE 5 (2010): 10074. 10.1371/journal.pone.0010074 PMC2852405 20404917

[123] H.‐L. Zhang , Z.‐N. Pan , J.‐Q. Ju , Y.‐M. Ji , Y. Wang , and S.‐C. Sun , “Formin INF2 Supplementation Alleviates Cytoskeleton‐Based Mitochondria Defects for Oocyte Quality under Obesity,” Free Radical Biology and Medicine 233 (2025): 250–263, 10.1016/j.freeradbiomed.2025.04.003. 40180021

[124] A. L. Elías‐López , O. Vázquez‐Mena , and A. N. Sferruzzi‐Perri , “Mitochondrial Dysfunction in the Offspring of Obese Mothers and It's Transmission through Damaged Oocyte Mitochondria: Integration of Mechanisms,” Biochimica et Biophysica Acta (BBA)—Molecular Basis of Disease 1869 (2023): 166802. 37414229 10.1016/j.bbadis.2023.166802

[125] Y. He , S. Deng , Y. Wang , et al., “Evaluation of Ovarian Stiffness and Its Biological Mechanism Using Shear Wave Elastography in Polycystic Ovary Syndrome,” Scientific Reports 15 (2025): 585, 10.1038/s41598-024-84338-8. 39747947 PMC11695736

[126] M. Contestabile , I. Marzi , C. Mangione , F. Franzoni , P. G. Artini , and S. Daniele , “Endometriosis and Oocyte Quality: Morphological Alterations, Developmental Competence, and Modifiable Strategies for Reproductive Longevity,” Cells 15 (2026): 296. 41677659 10.3390/cells15030296 PMC12897013

[127] D. A. Vaughan and D. Sakkas , “Sperm Selection Methods in the 21st Century,” Biology of Reproduction 101 (2019): 1076–1082, 10.1093/biolre/ioz032. 30801632

[128] R. Nuñez‐Calonge , N. Santamaria , T. Rubio , and J. M. Moreno , “Making and Selecting the Best Embryo in In Vitro Fertilization,” Archives of Medical Research 55 (2024): 103068, 10.1016/j.arcmed.2024.103068. 39191078

[129] S. Ozturk , “Selection of Competent Oocytes by Morphological Criteria for Assisted Reproductive Technologies,” Molecular Reproduction and Development 87 (2020): 1021–1036, 10.1002/mrd.23420. 32902927

[130] Y. Lemseffer , M.‐E. Terret , C. Campillo , and E. Labrune , “Methods for Assessing Oocyte Quality: A Review of Literature,” Biomedicines 10 (2022): 2184. 36140285 10.3390/biomedicines10092184 PMC9495944

[131] D. Nikiforov , M. L. Grøndahl , J. Hreinsson , and C. Y. Andersen , “Human Oocyte Morphology and Outcomes of Infertility Treatment: a Systematic Review,” Reproductive Sciences 29 (2022): 2768–2785, 10.1007/s43032-021-00723-y. 34816375

[132] T. Ebner , M. Moser , M. Sommergruber , M. Puchner , R. Wiesinger , and G. Tews , “Developmental Competence of Oocytes Showing Increased Cytoplasmic Viscosity,” Human Reproduction 18 (2003): 1294–1298, 10.1093/humrep/deg232. 12773462

[133] H. G. Jeong , S. K. Kim , J. Lee , H. W. Youm , and B. C. Jee , “Injection Funnel Persistence Time and Oolemma Resistance during Intracytoplasmic Sperm Injection and Subsequent Embryo Development,” Journal of Obstetrics and Gynaecology Research 47 (2021): 3590–3597, 10.1111/jog.14943. 34288279

[134] J. Abadie , C. Roux , E. Piat , C. Filiatre , and C. Amiot , “Experimental Measurement of Human Oocyte Mechanical Properties on a Micro and Nanoforce Sensing Platform Based on Magnetic Springs,” Sensors and Actuators B: Chemical 190 (2014): 429–438, 10.1016/j.snb.2013.08.042.

[135] R. Sciorio , D. Miranian , and G. D. Smith , “Non‐Invasive Oocyte Quality Assessment,” Biology of Reproduction 106 (2022): 274–290, 10.1093/biolre/ioac009. 35136962

[136] Y. Zeng , B. Cai , C. Ding , and Y. Xu , “Future Perspectives of Non‐Invasive Techniques for Evaluating Oocyte and Embryo Quality,” The Innovation Medicine 2 (2024): 100055, 10.59717/j.xinn-med.2024.100055.

[137] J. P. Evans and D. N. Robinson , “Micropipette Aspiration of Oocytes to Assess Cortical Tension,” Mouse Oocyte Development: Methods and Protocols (Springer, 2018), 163–171. 10.1007/978-1-4939-8603-3_17 PMC6158774 29961265

[138] A. Pokrzywnicka , P. Sniadek , N. Malyszka , et al., “MEMS Cytometer for Porcine Oocyte Deformation Measurement,” Journal of Micromechanics and Microengineering 29 (2019): 095004, 10.1088/1361-6439/ab27f1.

[139] D. Azarkh , Y. Cao , J. Floehr , and U. Schnakenberg , “Viscoelastic Properties of Zona Pellucida of Oocytes Characterized by Transient Electrical Impedance Spectroscopy,” Biosensors 13 (2023): 442, 10.3390/bios13040442. 37185516 PMC10136587

[140] L. Barbier , B. Venzac , V. Nordhoff , and S. L. Gac , Applications of Microfluidic Systems in Biology and Medicine, ed. M. Tokeshi , (Springer Nature, 2024), 233–273.

[141] Z. Luo , S. Güven , I. Gozen , et al., “Deformation of a Single Mouse Oocyte in a Constricted Microfluidic Channel,” Microfluidics and Nanofluidics 19 (2015): 883–890, 10.1007/s10404-015-1614-0. 26696793 PMC4684828

[142] H. Saffari , S. Hajiaghalou , M. A. Hajari , H. Gourabi , D. Fathi , and R. Fathi , “Design and Fabrication of Aspiration Microfluidic Channel for Oocyte Characterization,” Talanta 254 (2023): 124098, 10.1016/j.talanta.2022.124098. 36462279

[143] T. Wu , Y. Wu , J. Yan , J. Zhang , and S. Wang , “Microfluidic Chip as a Promising Evaluation Method in Assisted Reproduction: a Systematic Review,” Bioengineering & Translational Medicine 9 (2024): 10625, 10.1002/btm2.10625. PMC10905557 38435817

[144] H. Iwayama and M. Yamashita , “Quantitative Evaluation of Intercellular Local Deformation of Human Oocytes during Piezo‐Assisted Intracytoplasmic Sperm Injection Using Video‐Based Motion Analysis,” F&S Science 2 (2021): 124–134, 10.1016/j.xfss.2021.01.004. 35559747

[145] P. Grasland‐Mongrain , A. Zorgani , S. Nakagawa , et al., “Ultrafast Imaging of Cell Elasticity with Optical Microelastography,” Proceedings of the National Academy of Sciences 115 (2018): 861–866, 10.1073/pnas.1713395115. PMC5798341 29339488

[146] A. Ajduk , T. Ilozue , S. Windsor , et al., “Rhythmic Actomyosin‐Driven Contractions Induced by Sperm Entry Predict Mammalian Embryo Viability,” Nature Communications 2 (2011): 417, 10.1038/ncomms1424. PMC3265380 21829179

[147] S. Morawiec , A. Ajduk , P. Stremplewski , B. F. Kennedy , and M. Szkulmowski , “Full‐Field Optical Coherence Microscopy Enables High‐Resolution Label‐Free Imaging of the Dynamics of Live Mouse Oocytes and Early Embryos,” Communications Biology 7 (2024): 1057, 10.1038/s42003-024-06745-x. 39191989 PMC11349948

[148] F. Wang , S. Hao , K. Park , A. Ahmady , and C. Zhou , “Label‐Free Evaluation of Mouse Embryo Quality Using Time‐Lapse Bright Field and Optical Coherence Microscopy,” Communications Biology 8 (2025): 612, 10.1038/s42003-025-08044-5. 40234728 PMC12000469

[149] Y. Du , Y. Chen , S. Zhang , et al., “Mechanical Characterization and Modelling of Subcellular Components of Oocytes,” Micromachines 13 (2022): 1087, 10.3390/mi13071087. 35888904 PMC9319074

[150] S. Lamont , J. Fropier , J. Abadie , et al., “Profiling Oocytes with Neural Networks from Images and Mechanical Data,” Journal of the Mechanical Behavior of Biomedical Materials 138 (2023): 105640, 10.1016/j.jmbbm.2022.105640. 36566663

[151] Y. Du , Y. Cai , Z. Yang , K. Gao , M. Sun , and X. Zhao , “Modeling and Validation of Oocyte Mechanical Behavior Using AFM Measurement and Multiphysics Simulation,” Sensors 25 (2025): 5479, 10.3390/s25175479. 40942919 PMC12431014

[152] D. Meyer , J. Kort , C. H. Chen , et al., “Development and Evaluation of a Usable Blastocyst Predictive Model Using the Biomechanical Properties of Human Oocytes,” PLoS ONE 19 (2024): 0299602, 10.1371/journal.pone.0299602. PMC11065297 38696439

[153] E. Borges Jr. , D. Braga , M. D. Collado , et al., “Artificial Intelligence–Driven Oocyte Assessment for Predicting Blastulation and High‐Quality Blastocyst Formation in Severe Male Factor Infertility,” F&S Science 6 (2025): 426–432, 10.1016/j.xfss.2025.07.003. 40675554

[154] D. Cimadomo , V. Badajoz , M. Hebles , et al., “Artificial Intelligence‐Based Donor Oocyte Quality Assessment Moderately Improves the Prediction of Blastocyst Development: a First Step towards Higher Personalization in the Management of Egg Donation Treatments,” Human Reproduction 40 (2025): 1886–1892, 10.1093/humrep/deaf153. 40759152

[155] D. Adhikari , I. Lee , W. S. Yuen , and J. Carroll , “Oocyte Mitochondria—Key Regulators of Oocyte Function and Potential Therapeutic Targets for Improving Fertility,” Biology of Reproduction 106 (2022): 366–377, 10.1093/biolre/ioac024. 35094043

[156] H. Wang , Z. Huang , X. Shen , et al., “Rejuvenation of Aged Oocyte through Exposure to Young Follicular Microenvironment,” Nature Aging 4 (2024): 1194–1210, 10.1038/s43587-024-00697-x. 39251866

[157] D. Saha , S. Manshaei , T. Cavazza , et al., “Restoring Shugoshin 1 Reduces Chromosome Errors in Human Eggs,” bioRxiv, preprint (2026), 2026–3001, 10.64898/2026.01.08.698387.

[158] T. Umehara , Y. E. Winstanley , E. Andreas , et al., “Female Reproductive Life Span Is Extended by Targeted Removal of Fibrotic Collagen from the Mouse Ovary,” Science Advances 8 (2022): abn4564, 10.1126/sciadv.abn4564. PMC9205599 35714185

[159] W. Ju , S. Zhao , D. Li , J. Zhang , S. Xiang , and F. Lian , “Targeting Programmed Cell Death with Natural Products: a Potential Therapeutic Strategy for Diminished Ovarian Reserve and Fertility Preservation,” Frontiers in Pharmacology 16 (2025): 1546041, 10.3389/fphar.2025.1546041. 40510420 PMC12158948

[160] J. Tesarik and R. M. Tesarik , “Clinical Strategies for Counteracting Human Ovarian Aging: Molecular Background, Update, and Outlook,” International Journal of Molecular Sciences 26 (2025): 11973, 10.3390/ijms262411973. 41465398 PMC12732613

[161] Q. Zhuan , J. Li , X. Du , et al., “Antioxidant Procyanidin B2 Protects Oocytes against Cryoinjuries via Mitochondria Regulated Cortical Tension,” Journal of Animal Science and Biotechnology 13 (2022): 95, 10.1186/s40104-022-00742-y. 35971139 PMC9380387

[162] M. W. Jurema and D. Nogueira , “In Vitro Maturation of Human Oocytes for Assisted Reproduction,” Fertility and Sterility 86 (2006): 1277–1291, 10.1016/j.fertnstert.2006.02.126. 16996508

[163] X. Du , J. Li , Q. Zhuan , et al., “Artificially Increasing Cortical Tension Improves Mouse Oocytes Development by Attenuating Meiotic Defects during Vitrification,” Frontiers in Cell and Developmental Biology 10 (2022): 876259, 10.3389/fcell.2022.876259. 35399525 PMC8987233

[164] B. G. Hosu , S. F. Mullen , J. K. Critser , and G. Forgacs , “Reversible Disassembly of the Actin Cytoskeleton Improves the Survival Rate and Developmental Competence of Cryopreserved Mouse Oocytes,” PLoS One 3 (2008): 2787. 10.1371/journal.pone.0002787 PMC2467491 18665248

[165] C. J. Chan , C. Bevilacqua , and R. Prevedel , “Mechanical Mapping of Mammalian Follicle Development Using Brillouin Microscopy,” Communications Biology 4 (2021): 1133, 10.1038/s42003-021-02662-5. 34580426 PMC8476509

[166] J. Vasse , J. Fiscus , E. Fraison , B. Salle , L. David , and E. Labrune , “Biomechanical Properties of Ovarian Tissue and Their Impact on the Activation of Follicular Growth: a Narrative Review,” Reproductive BioMedicine Online 50 (2025): 104450, 10.1016/j.rbmo.2024.104450. 39919556

[167] H. Wang and L. Yang , “Ovarian Mechanobiology: Understanding the Interplay between Mechanics and Follicular Development,” Cells 14 (2025): 355, 10.3390/cells14050355. 40072084 PMC11898978

[168] T. Kawai and M. Shimada , “Changes in Ovarian Hardness and Elasticity Affect the Development and Function of Secondary Follicles,” Scientific Reports 16 (2026): 8837, 10.1038/s41598-026-39396-5. 41688648 PMC12982486

[169] S. Pietroforte and F. Amargant , “Increased Stiffness Mimicking Ovarian Aging Induces a Fibroinflammatory Response in Follicles and Impairs Oocyte Quality,” Reproduction 171 (2026): xaaf026, 10.1093/reprod/xaaf026.41575513

[170] F. Amargant , S. L. Manuel , Q. Tu , et al., “Ovarian Stiffness Increases with Age in the Mammalian Ovary and Depends on Collagen and Hyaluronan Matrices,” Aging Cell 19 (2020): 13259, 10.1111/acel.13259. PMC7681059 33079460

[171] C. H. de Koning , J. McDonnell , A. P. N. Themmen , F. H. de Jong , R. Homburg , and C. B. Lambalk , “The Endocrine and Follicular Growth Dynamics throughout the Menstrual Cycle in Women with Consistently or Variably Elevated Early Follicular Phase FSH Compared with Controls,” Human Reproduction 23 (2008): 1416–1423. 18375407 10.1093/humrep/den092

[172] P. Reddy , L. Liu , D. Adhikari , et al., “Oocyte‐Specific Deletion of Pten Causes Premature Activation of the Primordial Follicle Pool,” Science 319 (2008): 611–613, 10.1126/science.1152257. 18239123

[173] C. Farquhar , J. Brown , and J. Marjoribanks , “Laparoscopic Drilling by Diathermy or Laser for Ovulation Induction in Anovulatory Polycystic Ovary Syndrome,” The Cochrane Database of Systematic Reviews 6 (2012): CD001122. 10.1002/14651858.CD001122.pub4 22696324

[174] H. A. Hashim , “Response to: Predictors of Success of Laparoscopic Ovarian Drilling in Women with Polycystic Ovary Syndrome; an Evidence‐Based Approach,” Archives of Gynecology and Obstetrics 291 (2015): 717–718, 10.1007/s00404-015-3637-x. 25638451

[175] Z. Lin , Y. Li , Y. Zhao , et al., “Antifibrotic Drug Finerenone Restores Fertility in Premature Ovarian Insufficiency,” Science 391 (2026): adz4075, 10.1126/science.adz4075. 41643022

